# Kin2, the Budding Yeast Ortholog of Animal MARK/PAR-1 Kinases, Localizes to the Sites of Polarized Growth and May Regulate Septin Organization and the Cell Wall

**DOI:** 10.1371/journal.pone.0153992

**Published:** 2016-04-20

**Authors:** Si-Min Yuan, Wen-Chao Nie, Fei He, Zhi-Wen Jia, Xiang-Dong Gao

**Affiliations:** 1 Department of Microbiology, College of Life Sciences, Wuhan University, Wuhan, China; 2 Hubei Provincial Cooperative Innovation Center of Industrial Fermentation, Wuhan, China; University of Tokyo, JAPAN

## Abstract

MARK/PAR-1 protein kinases play important roles in cell polarization in animals. Kin1 and Kin2 are a pair of MARK/PAR-1 orthologs in the budding yeast *Saccharomyces cerevisiae*. They participate in the regulation of secretion and ER stress response. However, neither the subcellular localization of these two kinases nor whether they may have other cellular functions is clear. Here, we show that Kin2 localizes to the sites of polarized growth in addition to localization on the plasma membrane. The localization to polarity sites is mediated by two targeting domains—TD1 and TD2. TD1 locates in the N-terminal region that spans the protein kinase domain whereas TD2 locates in the C-terminal end that covers the KA1 domain. We also show that an excess of Kin2 activity impaired growth, septin organization, and chitin deposition in the cell wall. Both TD1 and TD2 contribute to this function. Moreover, we find that the C-terminal region of Kin2 interacts with Cdc11, a septin subunit, and Pea2, a component of the polarisome that is known to play a role in septin organization. These findings suggest that Kin2 may play a role in the regulation of the septin cytoskeleton and the cell wall. Finally, we show that the C-terminal region of Kin2 interacts with Rho3, a Rho GTPase, whereas the N-terminal region of Kin2 interacts with Bmh1, a 14-3-3 protein. We speculate that Kin2 may be regulated by Bmh1, Rho3, or Pea2 *in vivo*. Our study provides new insight in the localization, function, and regulation of Kin2.

## Introduction

The budding yeast *Saccharomyces cerevisiae* undergoes polarized growth during bud emergence and bud growth. This process relies on the polarized delivery of secretory vesicles to the growing bud tip and the rapid remodeling of the cell wall [1, 2]. A number of proteins that include Kin2 and its paralog Kin1 play a role in the regulation of polarized growth.

Kin1 and Kin2 are a pair of closely related serine/threonine protein kinases in *S*. *cerevisiae* [3–5]. They share 50% of sequence identity along their entire length and 90% of identity in the kinase domain [3]. Kin1 and Kin2 are known to positively regulate secretion, a process crucial for bud emergence and growth, as high-copy *KIN2* or *KIN1* suppressed the growth defect of secretion-defective *rho3-V51*, *cdc42-6*, *sec1-1*, *sec2-41*, *sec4-P48*, *sec10-2*, and *sec15-1* mutants [6, 7]. Moreover, heterologous overexpression of Kin2 in the yeast *Pichia pastoris* enhanced the secretion of the Fab fragment of a monoclonal antibody [8]. Kin1 and Kin2 are thought to regulate secretion by increasing the level and, possibly, the activity of Sec9 in the cytosol since *GAL*-driven overexpression of Kin2 and Kin1 caused the release of a fraction of Sec9 from the plasma membrane into the cytosol and induced the phosphorylation of cytosolic Sec9 [7]. Recently, Kin1 and Kin2 were reported to play a role in the unfolded protein response (UPR) in the endoplasmic reticulum (ER), a process that resolves the unfolded and misfolded proteins during ER stress, by regulating the targeting, splicing, and translation of *HAC1* mRNA [9]. Interestingly, Kin2’s functions in secretion and the ER stress response are both solely mediated by the protein kinase domain but not the C-terminal region [7, 9].

Orthologs of Kin1 and Kin2 are widespread in eukaryotes from yeast to humans and together they comprise the MARK/PAR-1/Kin1 family of protein kinases [10]. Some of the best studied orthologs include PAR-1 in the nematode *Caenorhabditis elegans* [11], MARKs (microtubule-associated protein/microtubule affinity regulating kinases) in mammals [12, 13]. These proteins play important roles in the regulation of cell polarity in animal embryos, epithelial cells, and neurons. For example, PAR-1 is essential for the establishment of anterior-posterior polarity in early *C*. *elegans* embryos [11]. MARK2 is required for the establishment of neuronal polarity and the growth of neurites in mice [14].

SpKin1, the sole fission yeast ortholog of Kin1 and Kin2, is involved in the control of polarized growth. Cells lacking SpKin1 showed reduced growth at 37°C and displayed an enlarged new cell end. Moreover, the cells displayed a defect in cell separation and had defects in the cell wall [15–17]. In contrast to *S*. *pombe* Sp*kin1*Δ mutants, deletion of either *KIN1* or *KIN2* or both in *S*. *cerevisiae* did not produce any detectable phenotype in growth or cell morphology [4, 5]. Thus, apart from roles in secretion and ER stress response, it is not known what other cellular functions Kin2 and Kin1 may have in budding yeast. In this study, we investigated the subcellular localization and cellular function of Kin2. We show that Kin2 localized to the sites of polarized growth and a higher dose of Kin2 affected septin organization and cell wall. We also show that Kin2 interacted with the septin subunit Cdc11, the polarisome component Pea2, Rho3 GTPase, and the 14-3-3 protein Bmh1. These findings provided new insight in Kin2’s functions and regulation.

## Results

### Kin2 localizes to the sites of polarized growth during bud growth

Biochemical fractionation data suggested that Kin2 localizes to the cytoplasmic face of the plasma membrane [18], implying that Kin2 may regulate exocytosis from the plasma membrane. A recent study using a GFP-Kin2 fusion construct, however, showed that Kin2 localizes to some punctated dots in the cytoplasm, but not to the plasma membrane [9]. This new observation poses a challenge to explain Kin2’s role in exocytosis. To resolve this discrepancy, we re-examined Kin2’s localization. We expressed the N-terminally GFP-tagged GFP-Kin2 under the control of Kin2’s endogenous promoter. GFP-Kin2 was barely visible in yeast cells when expressed on a low-copy centromere plasmid. After switching to a high-copy plasmid vector, which may increase the expression level of GFP-Kin2, GFP fluorescence was readily detected. This GFP-Kin2 construct was functional in regulating exocytosis since it suppressed the temperature-sensitive growth defects of *sec1-1* and *sec2-41* mutants on high-copy plasmids (data not shown).

**Fig 1:**
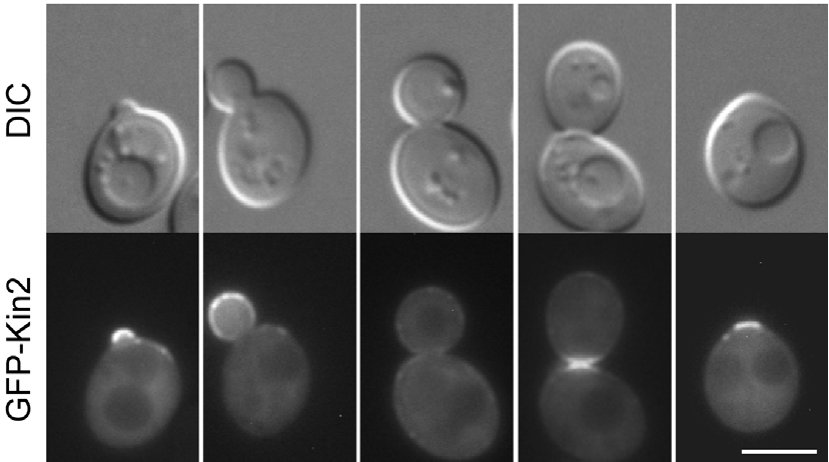
GFP-Kin2 localization during bud development. Cells of yeast strain YEF473A carrying plasmid pKG21-KIN2 were grown on SC-Ura plate at 30°C for 16 hr and examined for GFP fluorescence. Bar, 5 μm.

**Fig 2:**
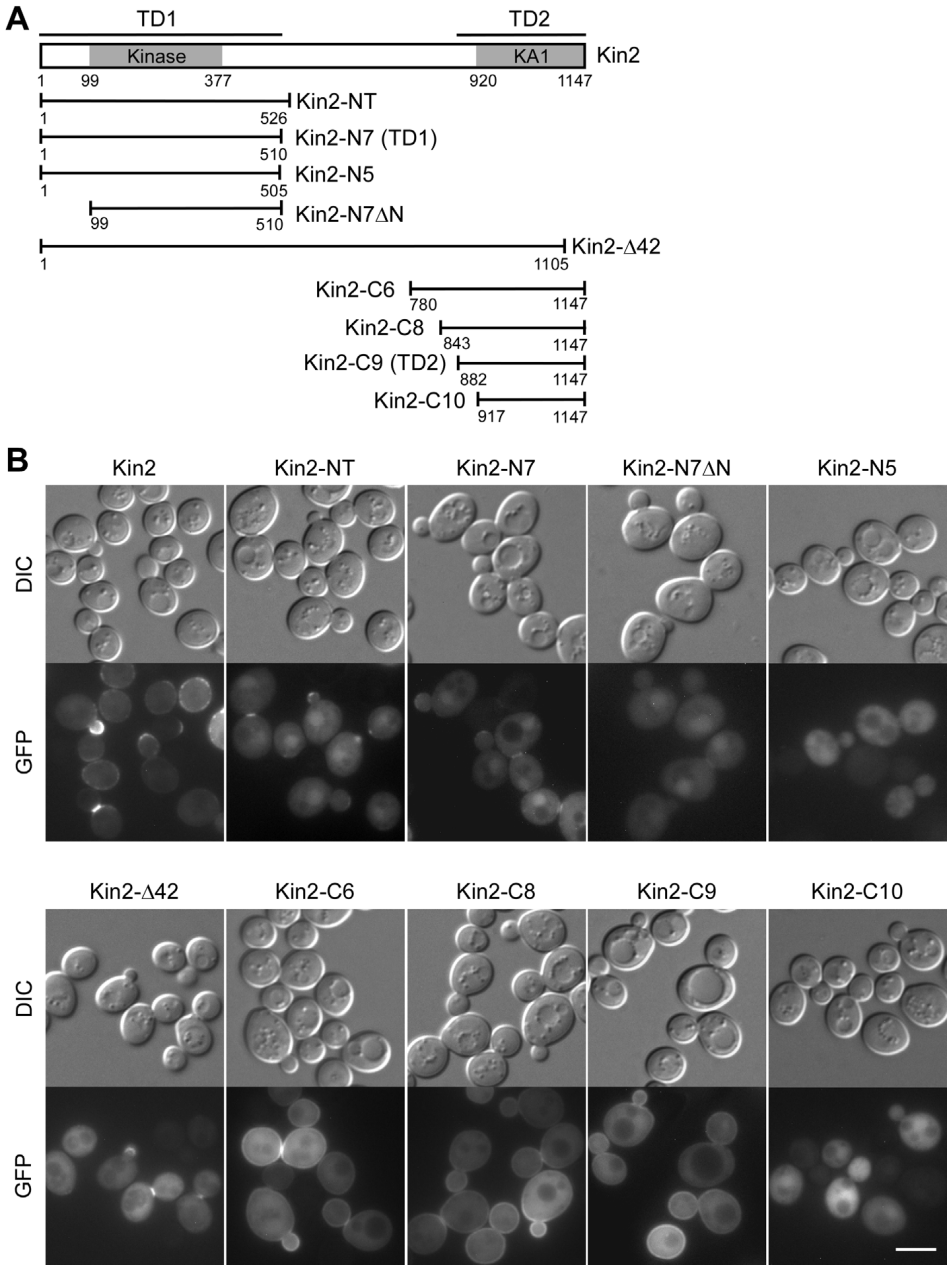
Kin2 contains two distinct targeting domains for localization. **(A)** Schematic representation of Kin2’s domains and the Kin2 segments used in the study. **(B)** Localization of GFP-Kin2 and GFP-Kin2 segments. Cells of strain YEF473A carrying pUG36-KIN2 and pUG36-KIN2 segments were grown on SC-Ura plate at 30°C for 16 hr and then examined for GFP fluorescence. Bar, 5 μm.

As shown in Fig 1, GFP-Kin2 localized to the sites of polarized growth in a cell cycle-dependent manner. GFP-Kin2 was highly enriched on the bud cortex at the small-budded stage as 39% of small-budded cells (*n* = 606) displayed an enrichment of GFP-Kin2 on the bud cortex. The remaining cells either displayed an even distribution of fluorescence in the bud and mother cell cortex (1%) or lacked visible GFP signal in the cells (60%). The enrichment of GFP-Kin2 at the bud cortex persisted at the medium-budded stage, but gradually diminished as the bud grew larger. 14% of medium-budded cells (*n* = 271) still displayed an enrichment of GFP-Kin2 on the bud cortex whereas 20% of cells showed an even distribution in the bud and mother cell cortex (Fig 1, see the middle cell). The remaining cells (66%) lacked visible GFP signal in the cells. Around the time of cytokinesis, GFP-Kin2 relocated to the mother-bud neck. We observed that 26% of large-budded cells (*n* = 324) displayed bright GFP fluorescence at the bud neck (Fig 1, see the second cell from right), whereas 2% of large-budded cells did not displayed an enrichment at the bud neck. The remaining cells (72%) lacked visible GFP signal in the cells. After the daughter cell and the mother separated, GFP-Kin2 remained at the old bud neck for a while and then gradually disappeared. In some cells that had already entered the next round of budding, GFP-Kin2 could still be seen at the old bud neck (Fig 1, see the left two cells. The old bud neck locates next to the newly formed bud). The polarized distribution of Kin2 implies that Kin2 may play a role in polarized cell growth. In addition to the polarized localization, GFP-Kin2 also localized to the entire cell cortex (Fig 1, see the right three cells). This finding is in agreement with the observation that Kin2 associates with the plasma membrane [18]. Interestingly, cortical GFP-Kin2 was markedly concentrated at some spots or patches whose identity is not clear (Fig 1, see the middle cell, and Fig 2B, see Kin2).

### Two distinct domains mediate Kin2’s localization

Elbert *et al*. showed that the N-terminal region a.a. 1–526 of Kin2 is the functional determinant for the multicopy suppression of several late secretory *sec* mutants by Kin2 [7]. To gain insight into the mechanism by which the N-terminal region of Kin2 functions, we asked whether it could localize properly. We found that this region, denoted as Kin2-NT (Fig 2A) [7], lacked the general plasma membrane localization like full-length Kin2 did. However, Kin2-NT did localize to the sites of polarized growth such as the bud tip in small-budded cells and the bud neck in large-budded cells (Fig 2B), suggesting that Kin2 may function at these sites. The polarity-site-targeting function of Kin2-NT appears to be mediated by the region a.a. 1–510 since this region (named Kin2-N7) localized to these sites (Fig 2B). Further truncation at the C-terminus completely abolished bud tip and bud neck localization (Fig 2B, see Kin2-N5). The removal of the first 98 amino acids also eliminated bud tip localization in small-budded cells and nearly abolished bud neck localization in large-budded cells (Fig 2B, see Kin2-N7ΔN). We thus named the region a.a. 1–510 targeting domain 1 (TD1).

Although the TD1 domain of Kin2 localized to polarized growth sites, its bud tip localization was markedly reduced compared to that of full-length Kin2 (Fig 2B, see Kin2-NT). Moreover, it lacked a general association with the plasma membrane as full-length Kin2 did, suggesting that there might be a second targeting domain within the C-terminal half of Kin2. Kin2 contains a KA1 domain (Kinase-Associated domain 1) (a.a. 920–1147) at the C-terminus that is highly conserved in members of the MARK/PAR-1/Kin1 family (Fig 2A) [19] (Note: The KA1 domain here is the 228 amino-acid-long version but not the short 43 amino acid sequence defined by the Pfam database). We found that the removal of the last 42 amino acids of the KA1 domain completely destroyed Kin2’s plasma membrane localization while its localization to the polarity sites remained (Fig 2B, see Kin2-Δ42), suggesting that the KA1 domain is essential for targeting Kin2 to the plasma membrane. Despite of its critical role in cortex targeting, the KA1 domain alone was not sufficient for localization to the plasma membrane (Fig 2B, see Kin2-C10). It is likely that upstream sequence may be required. By examining Kin2-C segments with upstream sequences of various length for localization, we found that Kin2-C6 (a.a. 780–1147), Kin2-C8 (a.a. 843–1147), and Kin2-C9 (a.a. 882–1147), three Kin2-C segments longer than Kin2-C10 (a.a. 917–1147), all localized to the plasma membrane (Fig 2B). In addition to plasma membrane targeting, Kin2-C6 and Kin2-C8 also showed a modest enrichment on the bud cortex in small-budded cells and at the bud neck in large-budded cells, which resembles the localization of full-length Kin2. Kin2-C9 also showed a slight enrichment on the bud cortex in a few small-budded cells. Thus, the region a.a. 882–1147 that contains the KA1 domain appears to be the second polarity-site-targeting domain. We named it targeting domain 2 (TD2). This result indicates that the C-terminal half of Kin2 could also concentrate at the sites of polarized growth. All these GFP-fused Kin2 segments were expressed well in the cells as determined by immunoblotting with an anti-GFP antibody (S1 Fig).

Together, our results show that Kin2 contains two distinct domains for targeting to the sites of polarized growth. The C-terminal TD2 domain also mediates an association with the plasma membrane.

### Kin2’s localization to the polarity site is critical for its function in exocytosis

**Fig 3:**
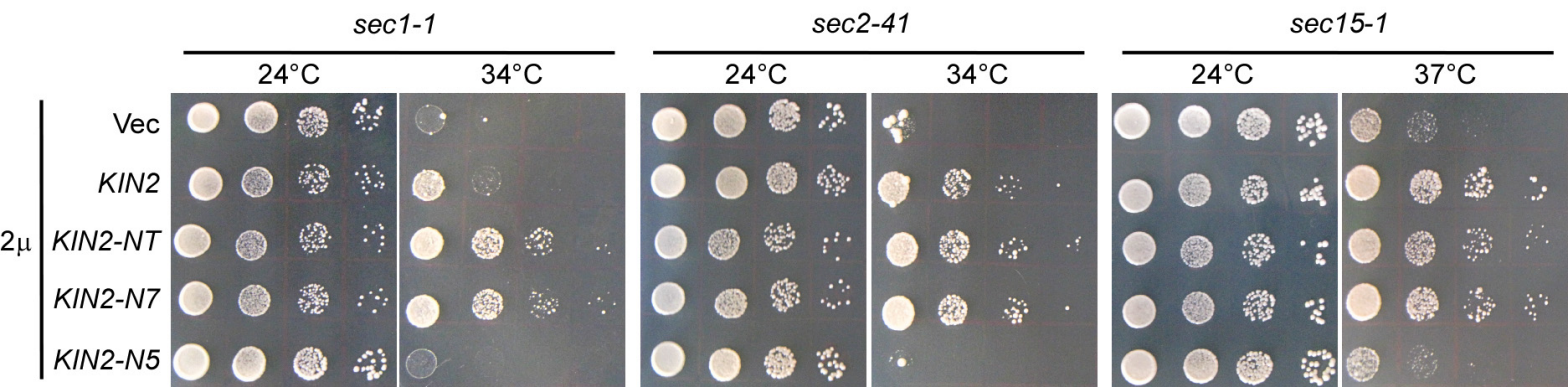
Kin2’s localization to the polarity site is essential for its multicopy suppression of late secretory mutants. Cells of strains JGY27B (*sec1-1*), JGY28B (*sec2-41*), JGY82A (*sec15-1*) carrying pRS426 (Vec), pRS426-KIN2, pRS426-KIN2-NT, pRS426-KIN2-N7, and pRS426-KIN2-N5 were spotted on SC-Ura plate at 1:10 serial dilution and incubated at permissive (24°C) and their respective restrictive temperatures. Pictures were taken after 4 days.

Kin2’s kinase activity is known to be critical for the suppression of several temperature-sensitive *sec* mutants [7]. We asked if the localization to the sites of polarized growth is also required for Kin2’s function in exocytosis. We found that multicopy expression of Kin2-NT (a.a. 1–526), the previously known functional segment, as well as Kin2-N7 (a.a. 1–510), the TD1 domain itself, both suppressed the growth defect of *sec1-1*, *sec2-41*, and *sec15-1* mutants. In contrast, multicopy expression of Kin2-N5 (a.a. 1–505), a Kin2 segment that completely lacks polarity-site localization due to the lack of an intact TD1 domain, did not suppress the growth defect of these *sec* mutants (Fig 3). This result suggests that Kin2’s function in exocytosis not only requires its kinase activity but also its localization to the sites of polarized growth.

### Overexpression of Kin2 affects septin organization and cell wall

**Fig 4:**
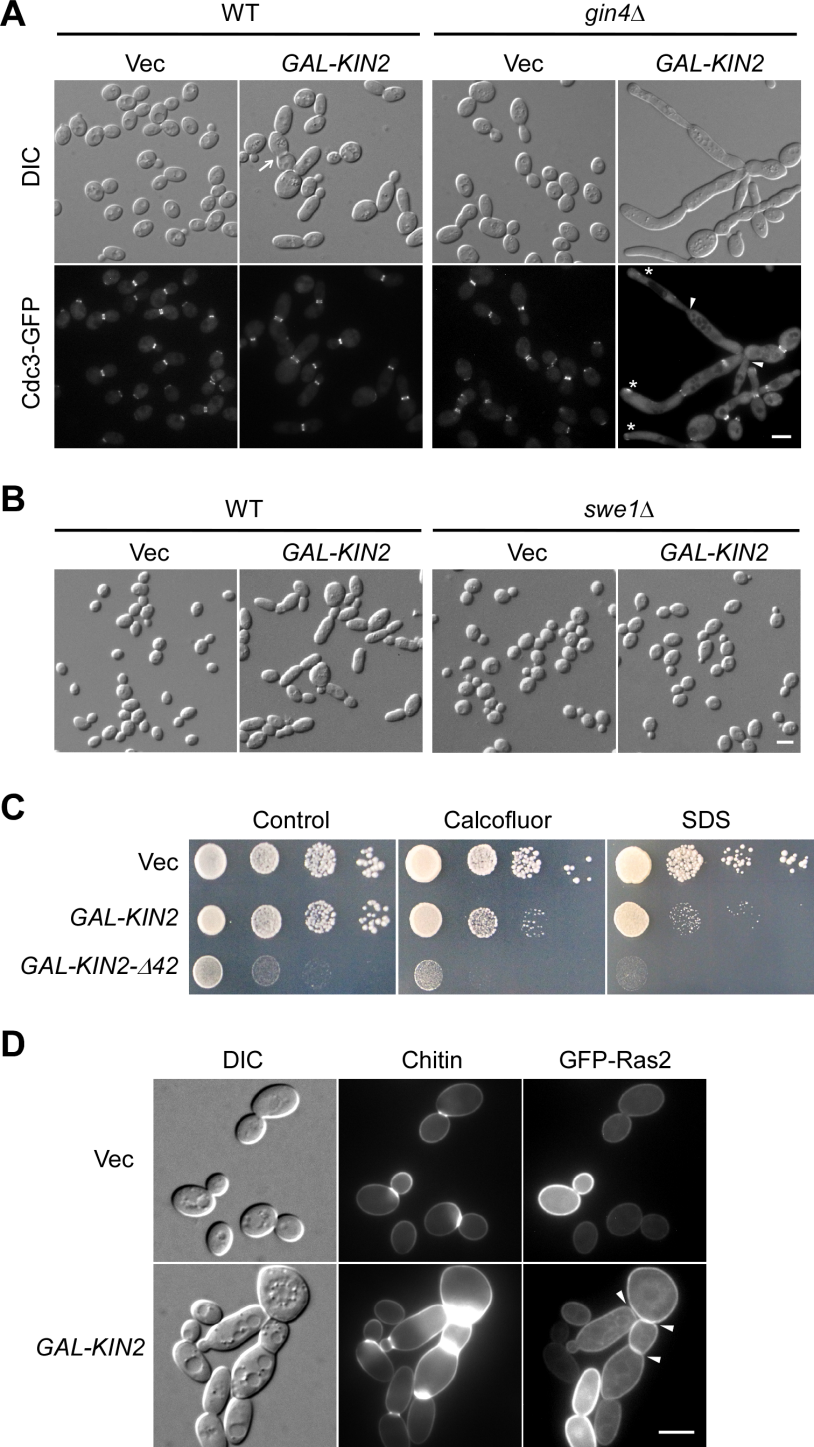
Overexpression of Kin2 affects septin organization and cell wall organization. **(A)** Cell morphology and Cdc3-GFP localization of cells overexpressing Kin2. Cells of strain YEF473A (WT) and YEF1238 (*gin4*Δ) with integrated *CDC3-GFP*:*LEU2* carrying pEGKT316 (Vec) or pEGKT316-KIN2 were grown on SRG-Ura plate at 30°C for 20 hr before imaging. **(B)** Morphology of wild-type and *swe1*Δ cells overexpressing Kin2. Cells of strain YEF473A (WT) and JGY2030 (*swe1*Δ) carrying pEGKT316 (Vec) or pEGKT316-KIN2 were grown on SRG-Ura plate. **(C)** Cells overexpressing Kin2 or Kin2-Δ42 are sensitive to calcofluor white and SDS. YEF473A cells carrying pEGKT316 (Vec), pEGKT316-KIN2, pEGKT316-KIN2-Δ42 plasmids were grown on SRG-Ura medium (control), SRG-Ura medium with 5 μg/ml calcofluor white, and SRG-Ura medium with 40 μg/ml SDS at 30°C. Pictures were taken after 6 days. **(D)** Chitin distribution and GFP-Ras2 localization. Cells of strain YEF473A carrying plasmids pRS315-GFP-RAS2/pEGKT316 (Vec) or pRS315-GFP-RAS2/pEGKT316-KIN2 were grown on SRG-Leu-Ura plate at 30°C for 20 hr. Chitin was stained before imaging. Bars, 5 μm.

Previous studies have shown that deletion of either *KIN1* or *KIN2* or both did not impair growth or secretion [4, 5, 7]. Similarly, *kin1*Δ *kin2*Δ cells in our strain background (S288C derived) also did not show any detectable defect in growth or bud development. The lack of phenotype in *kin1*Δ *kin2*Δ cells imposes a challenge for studying the cellular functions of Kin2. To gain insight into the functions of Kin2 in yeast cells, we wanted to examine the effect of Kin2 overexpression on growth and bud development. Kin2 was then overexpressed under the control of a galactose-inducible promoter on a low-copy plasmid. We found that Kin2 overexpression did not impair growth in wild-type cells. However, it affected morphogenesis as a small percentage of cells (7%, *n* = 624) displayed an elongated cell morphology (Fig 4A, left panel). Moreover, 8% of cells (*n* = 595) became multibudded (Fig 4A, left panel, arrow).

**Fig 5:**
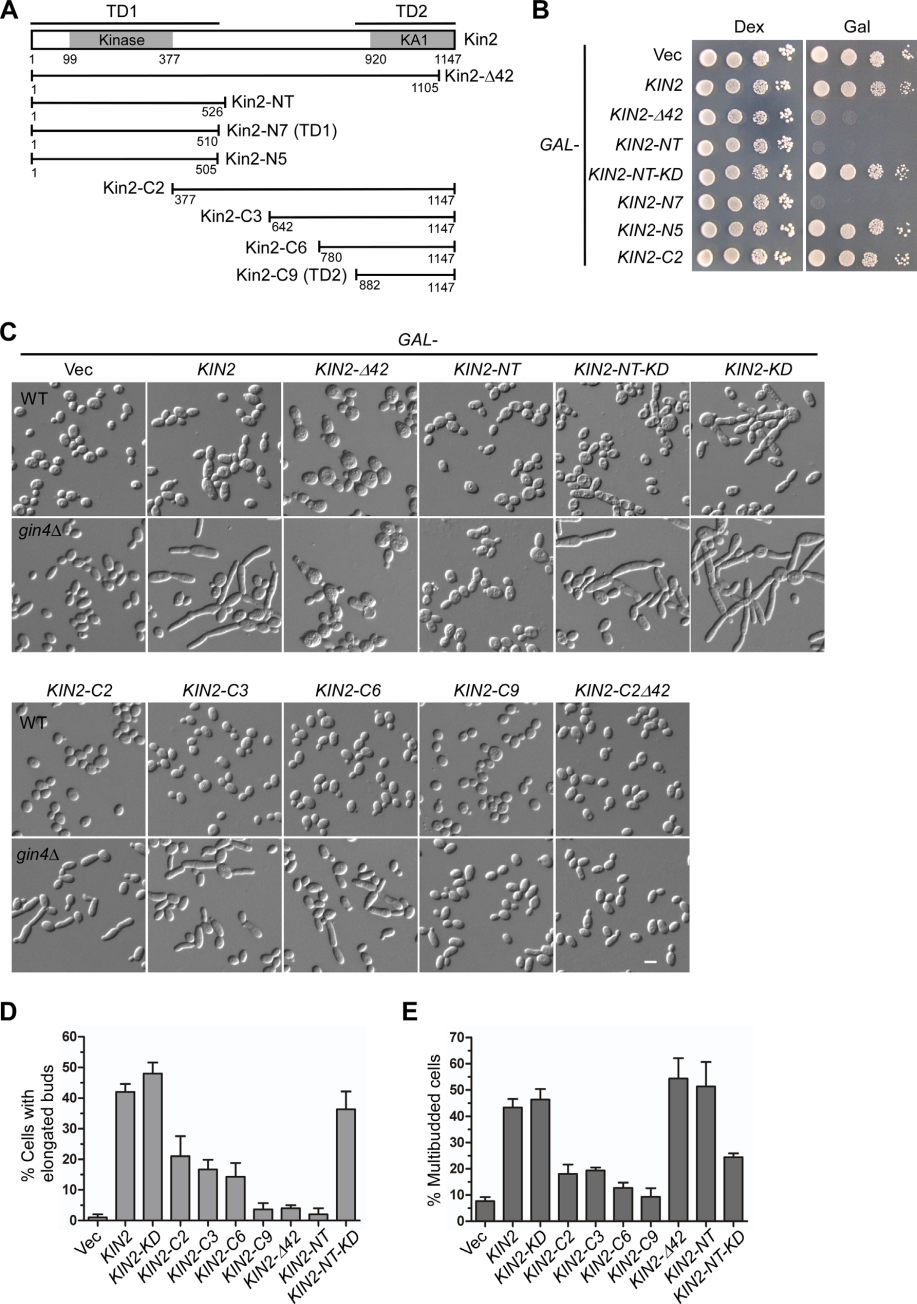
Phenotypes of cells overexpressing Kin2 segments. **(A)** Schematic representation of the Kin2 segments used in the study. **(B)** Cells of strain YEF473A carrying pEGKT316 (Vec), pEGKT316-KIN2, and pEGKT316-KIN2 segments were spotted on SC-Ura (Dex) plate and SRG-Ura (Gal) plate at 1:10 serial dilution and incubated at 30°C. Pictures were taken after 4 days. **(C)** Cells of strain YEF473A (WT) and YEF1238 (*gin4*Δ) carrying pEGKT316 (Vec), pEGKT316-KIN2, and pEGKT316-KIN2 segments were grown on SRG-Ura plate at 30°C for 20 hr before imaging. **(D-E)** Percentages of cells with elongated buds **(D)** and multibudded cells **(E)** in the population of budded *gin4*Δ cells overexpressing *KIN2* and *KIN2* segments as in **(C)** were quantitated. Bar, 5 μm.

Defects in septin organization in a number of yeast mutants, such as *gin4*Δ and *elm1*Δ mutants, trigger a Swe1-dependent G2 delay that inhibits the apical-to-isotropic switch of bud growth, generating an elongated-bud morphology [20–23]. The observation that Kin2 overexpression caused mild bud elongation in a small percentage of cells raises a possibility that high dose of Kin2 may affect septin organization. In support of this speculation, we found that Kin2 overexpression failed to induce bud elongation in *swe1*Δ cells (Fig 4B). However, when we examined septin organization (indicated by Cdc3-GFP) in cells overexpressing Kin2, we failed to detect any defect (Fig 4A, left panel, bottom row). This result suggests that the defect, if it does exist, may not be severe enough to be readily detected. We reasoned that the defect may become detectable in certain yeast mutants, which have already a less-well-organized septin cytoskeleton. To test this, we overexpressed Kin2 in *gin4*Δ mutant, a mutant in which septin organization is compromised to some extent. Gin4 is a protein kinase that binds and phosphorylates the septins and plays an important role in septin organization [22, 24]. As *gin4*Δ cells grown in synthetic SC medium at 30°C displayed a relatively normal morphology in which only 1% of cells (*n* = 1392) exhibited long buds and 7% of cells (*n* = 1344) were multibudded, we found that Kin2 overexpression in *gin4*Δ cells caused massive bud elongation and clumping. 42% of cells (*n* = 1332) displayed highly elongated buds and 43% of cells (*n* = 1046) were multibudded (Fig 4A, right panel, and Fig 5D and 5E). The cells displayed a defect in septin organization that was much stronger than that of control cells as Cdc3-GFP was often seen less concentrated at or absent from the bud neck (Fig 4A, right panel, bottom row, arrowheads). In the elongated buds, Cdc3-GFP was often mislocalized to a patch near the tip (Fig 4A, right panel, bottom row, asterisks). This result indicates that Kin2 overexpression perturbs septin organization and this effect is more pronounced in cells that had less-well-organized septin cytoskeleton.

Kin2 overexpression in wild-type cells also rendered the cells sensitive to calcofluor white (a fluorescent, chitin-binding, cell wall-damaging agent) and SDS (sodium dodecyl sulfate, a detergent that could damage the plasma membrane) (Fig 4C). A dominantly active mutant, Kin2-Δ42, had a similar effect upon overexpression except that its overexpression alone impaired growth. This result and the presence of multibudded cells suggest that Kin2 overexpression may cause a defect in the cell wall. We then stained the cells for chitin, a component of cell wall that is mainly deposited at the bud neck. Chitin staining with calcofluor revealed that chitin deposition at the bud neck was markedly increased in multibudded cells (Fig 4D), suggesting that cell wall organization was indeed abnormal. Next, we examined whether cytokinesis was completed in multibudded cells. By staining the plasma membrane with GFP-Ras2, we found that the cells at the base of a cell chain had already completed cytokinesis (Fig 4D, arrowheads), indicating that cell chains may result from a delay in cell-cell separation after cytokinesis, which in turn may result from enhanced chitin deposition at the bud neck. Together, these results indicate that Kin2 overexpression also affects cell wall organization.

Together, our results show that high dose of Kin2 alters septin organization and perturbs cell wall organization. Kin2 may normally play a role in these cellular processes.

### Roles of the two targeting domains in Kin2 overexpression-caused perturbation in septin organization

The phenotype of Kin2-overexpressing cells provides us an opportunity to investigate the roles of the two targeting domains in Kin2’s function. We thus overexpressed a series of Kin2’s N-terminal and C-terminal segments in wild type and *gin4*Δ cells and examined their effects on growth and cell morphology (Fig 5A). While overexpression of Kin2 and Kin2-C segments did not impair growth in wild-type cells, we found that overexpression of Kin2-NT or Kin2-N7 (TD1 domain) dramatically reduced growth (Fig 5B, see Kin2-C2 for example of Kin2-C segments). Overexpression of Kin2-Δ42, another dominantly active mutant, also dramatically impaired growth (Fig 5B). The growth inhibition depends on the kinase activity of Kin2 because the kinase-dead Kin2-NT-KD mutant (the lysine 128 residue was replaced by methionine) no longer impaired growth upon overexpression (Fig 5B). It also seems to depend on its proper localization to the polarity site since overexpression of Kin2-N5 (a.a. 1–505), a segment that is slightly shorter than Kin2-NT and does not localize to the site of polarized growth, did not impair growth (Fig 5B). Together, these results indicate that an excess of Kin2 activity is detrimental to cell growth.

Why was cell growth reduced upon Kin2-NT overexpression? We noticed that cells overexpressing Kin2-NT were normal in cell morphology except that 19% (*n* = 535) of cells were multibudded (Fig 5C). The cells did not seem to be arrested at a particular cell cycle stage. Methylene blue staining showed that the cells did not tend to die at a particular cell cycle stage (data not shown). Moreover, the addition of 1 M sorbitol, an agent that provides osmotic support, to the culture medium failed to relieve the growth inhibition (data not shown). It is not clear which key cellular process was affected in cells overexpressing Kin2-NT that had led to the significant reduction in growth rate.

As overexpression of full-length Kin2 caused dramatic bud elongation in *gin4*Δ cells, overexpression of Kin2-NT in *gin4*Δ cells did not induce bud elongation. However, overexpression of several Kin2-C segments caused bud elongation (Fig 5C, lower panel, and 5D). For example, 17% of *gin4*Δ cells (*n* = 1453) overexpressing Kin2-C3 (a.a. 642–1147) displayed elongated buds, suggesting that the C-terminal region of Kin2, but not the N-terminal region, may be responsible for causing bud elongation when Kin2 is overexpressed. The function of the C-terminal region of Kin2 in causing bud elongation requires an intact TD2 domain because while overexpression of Kin2-C2 (a.a. 377–1147) caused bud elongation in 21% of cells (*n* = 1602), overexpression of Kin2-C2Δ42 (a.a. 377–1105), a Kin2-C segment that lacks the last 42 a.a., no longer caused bud elongation (Fig 5C and 5D).

Although TD2 domain is critical for Kin2 overexpression-induced bud elongation, TD2 itself is not sufficient to induce bud elongation upon overexpression (Fig 5C, see Kin2-C9, and 5D). The region next to the TD2 domain, a.a. 780–881, is required for Kin2’s function in causing bud elongation because overexpression of Kin2-C6 (a.a. 780–1147), a Kin2-C segment longer than the TD2 domain, caused bud elongation in 14% of cells (*n* = 1479) (Fig 5C and 5D).

While overexpression of Kin2-NT did not induce bud elongation in *gin4*Δ cells, surprisingly, we noticed that overexpression of the kinase-dead Kin2-NT-KD mutant dramatically induced bud elongation as 36% of cells (*n* = 1236) had elongated buds. The magnitude of bud elongation is close to that of cells overexpressing full-length Kin2 (Fig 5C and 5D). The full-length kinase-dead Kin2-KD mutant also caused bud elongation in 48% of *gin4*Δ cells (*n* = 1145) upon overexpression (Fig 5C and 5D). Remarkably, the kinase-dead Kin2-KD and Kin2-NT-KD mutants could also induce dramatic bud elongation in wild-type cells upon overexpression whereas Kin2-C segments could not (Fig 5C, top panel). These results suggest that, besides the C-terminal region of Kin2, high dose of the N-terminal portion of Kin2 could also affect septin organization.

Another phenotype of Kin2 overexpression in *gin4*Δ cells is the dramatic increase of multibudded cells: 43% (*n* = 1046) for Kin2 overexpression compared to just 7% (*n* = 1344) for the control *gin4*Δ cells in the population of budded cells (Fig 5C and 5E). Both of the N-terminal region and the C-terminal region of Kin2 were involved in this function, with the N-terminal region playing a major role since 51% (*n* = 648) of cells overpressing Kin2-NT were multibudded whereas about 18% of cells overexpressiong Kin2-C2 or Kin2-C3 were multibudded (Fig 5C and 5E). The kinase activity of Kin2 contributed to this function to some extent but was not absolutely required (Fig 5E, compare Kin2-NT and Kin2-NT-KD).

Together, our results indicate that both of the N-terminal and the C-terminal regions of Kin2 are responsible for Kin2 overexpression-induced bud elongation, a defect caused by defective septin organization, and cell clumping. These two regions of Kin2 may normally interact with proteins implicated in septin assembly.

### The C-terminal region of Kin2 interacts with Cdc11, a septin subunit, and Pea2, a polarisome component involved in the regulation of septin assembly

**Fig 6:**
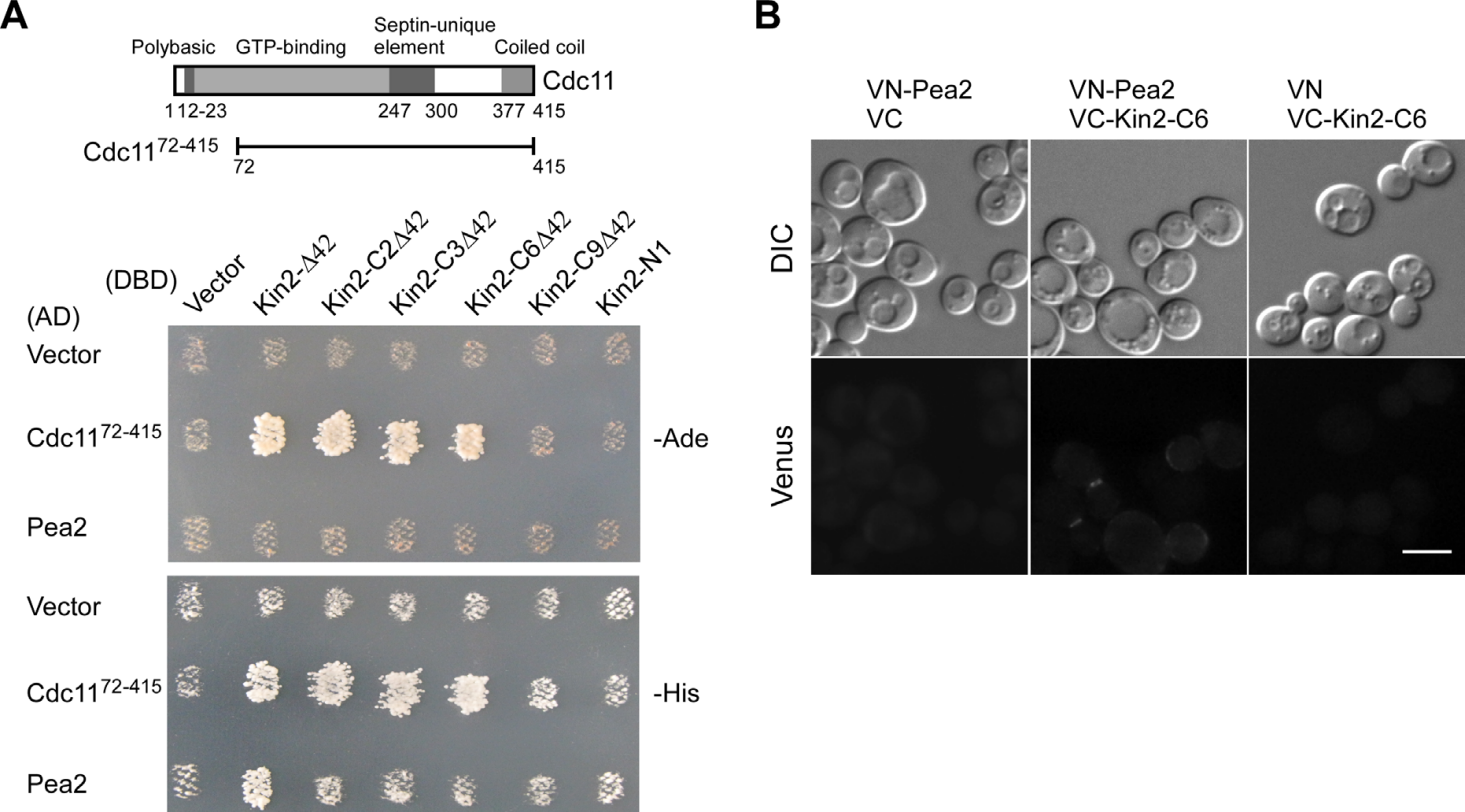
Kin2 interacts with Cdc11 and Pea2 via its C-terminal region. **(A)** Two-hybrid assay of the interaction of Kin2 segments with Cdc11 and Pea2. The assay was performed as described in *Materials and Methods*. pGBDU-C1 (DBD, vector) and pGBDU-KIN2 segments were used to pair with pOAD (AD, vector), pOAD-CDC11^72-415^ and pGAD-PEA2. Cells were grown on SC-Leu-Ura-Ade (-Ade) and SC-Leu-Ura-His (-His) plates at 30°C for 3 days. Growth indicates interaction between the DBD and AD fusion proteins. The domain structure of Cdc11 and Cdc11^72-415^ segment was depicted in the upper panel. **(B)** BiFC assay between Kin2-C6 and Pea2. Cells of strain JGY3088 (*kin1*Δ *kin2*Δ) carrying pVN1-PEA2/pVC1, pVN1-PEA2/pVC1-KIN2-C6, and pVN1/pVC1-KIN2-C6 pairs were grown on SC-His-Ura plate at 30°C for 16 hr. Green fluorescence was examined by fluorescence microscopy. Bar, 5 μm.

To explore how Kin2 may function in the cells, we wanted to identify proteins that interact with Kin2. From a two-hybrid screen with Kin2-Δ42 (the last 42 a.a. was removed to eliminate plasma-membrane targeting) as bait, we identified N-terminally truncated Cdc11 (the region a.a. 72–415) and Tos1 (the region a.a. 192–455 and a.a. 201–455). Cdc11 is one of five septin subunits expressed during vegetative growth [25]. Tos1 is a cell wall-associated protein that is homologous to glycosyl hydrolase family 16 [26, 27]. The interaction between Kin2 and Tos1 may have a role in cell-wall organization. We are particularly interested in the interaction between Kin2-Δ42 and Cdc11^72-415^ because it may provide a clue on how an excess of Kin2 may perturb septin organization. We found that Kin2 appears to interact with Cdc11^72-415^ via its C-terminal region, but not the N-terminal region since Kin2-C segments as short as Kin2-C6Δ42 (a.a. 780–1105) interacted with Cdc11^72-415^. However, we did not detect an interaction between the TD2 domain of Kin2 and Cdc11^72-415^ (Fig 6A, see Kin2-C9Δ42). This result suggests that Kin2 may interact with Cdc11^72-415^ via its C-terminal region a.a. 780–1105. We investigated whether Kin2 may interact with full-length Cdc11 in two-hybrid assay but failed to detect an interaction (data not shown).

We also identified an interaction between Kin2-Δ42 and Pea2, a component of the polarisome that is implicated in the assembly of septin filaments [28, 29], in the two-hybrid assay (Fig 6A). Although we failed to detect an interaction between the N-terminal and the C-terminal regions of Kin2 with Pea2 in the two-hybrid assay (Fig 6A, see Kin2-N1 and Kin2-CΔ42), we identified an interaction between Kin2-C6 (a.a. 780–1147) and Pea2 in the bimolecular fluorescence complementation (BiFC) assay (Fig 6B). The interaction appears to occur on the bud cortex. We also detected the interaction of Pea2 with Kin2-C segments longer than Kin2-C6 but not with the TD2 domain (Kin2-C9), which is shorter than Kin2-C6, in BiFC assay (data not shown). These results suggest that Kin2 may interact with Pea2 via the C-terminal region a.a. 780–1147.

Together, we identified an interaction of Kin2 with Cdc11 and Pea2, which are a septin subunit and a protein involved in septin assembly, respectively. Both of the two interactions appear to be mediated by the C-terminal region a.a. 780–1147 of Kin2. Since this region could disrupt septin organization upon overexpression in *gin4*Δ cells, the observed interactions may be physiologically significant in normal septin organization.

### Kin2 might be regulated by Rho3 and Bmh1 *in* *vivo*

**Fig 7:**
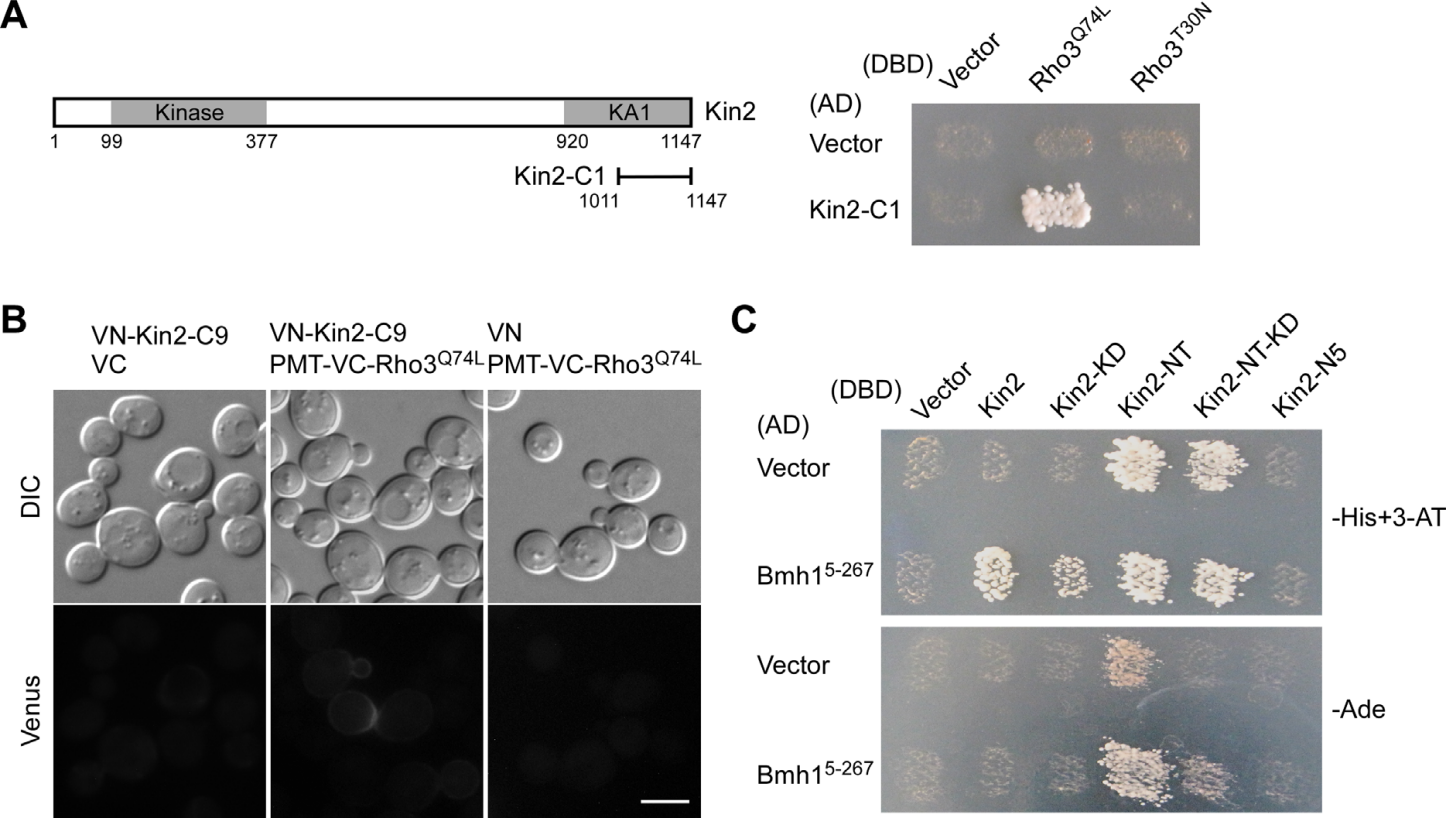
Kin2 interacts with Rho3 and Bmh1. **(A)** Two-hybrid assay of the interaction between Kin2-C1 and Rho3. The assay was performed as described in *Materials and Methods*. pGBDU-C1 (DBD, vector), pGBDU-RHO3^Q74L, ΔC^, and pGBDU-RHO3^T30N, ΔC^ were used to pair with pOAD (AD, vector) and pOAD-KIN2-C1. Cells were grown on SC-Leu-Ura-Ade plate at 30°C for 3 days. **(B)** BiFC assay between Kin2-C9 and Rho3. Cells of strain JGY3088 (*kin1*Δ *kin2*Δ) carrying pVN1-KIN2-C9/pVC1, pVN1-KIN2-C9/pVC1-PMT-RHO3^Q74L^, and pVN1/ pVC1-PMT-RHO3^Q74L^ pairs were grown on SC-His-Ura plate at 30°C for 16 hr. Green fluorescence was examined. Bar, 5 μm. **(C)** Two-hybrid assay of the interaction between Kin2 segments and Bmh1. pGBDU-C1 (DBD, vector) and pGBDU-KIN2 segments were used to pair with pOAD (AD, vector) and pOAD-BMH1^5-267^. Cells were grown on SC-Leu-Ura-His containing 3 mM 3-AT (-His+3-AT) and SC-Leu-Ura-Ade (-Ade) plates at 30°C for 3 days.

Kin2 contains a regulatory C-terminal domain that could bind to the N-terminal kinase domain and negatively regulates its activity [7]. Kin2’s activation is thought to require the opening up of the closed structure. So far, it is not clear which protein may play a role in the activation of Kin2. Rho3 is a Rho GTPase that plays a critical role in the regulation of polarized growth [30, 31]. In two-hybrid screen to identify proteins that interact with Rho3^Q74L^ [32], a constitutively active mutant of Rho3, one cDNA clone that encodes the C-terminal region, a.a. 1011–1147, of Kin2 was isolated. This Kin2 segment, denoted as Kin2-C1, consists of part of the KA1 domain (Fig 7A). The interaction of Kin2-C1 with Rho3 is GTP-dependent since Kin2-C1 preferentially interacted with the Rho3^Q74L^ mutant, but not with the constitutively inactive Rho3^T30N^ mutant in two-hybrid assay (Fig 7A). The interaction between Kin2-C1 and Rho3 is interesting because it may suggest that Kin2 and Rho3 may interact *in vivo*. We explored this possibility by using the two-hybrid assay but failed to detect an interaction between full-length Kin2 and Rho3^Q74L^ (data not shown).

We also examined the interaction using BiFC assay. Since Rho3 is palmitoylated at the cysteine 5 residue and this modification is important for Rho3’s localization to the plasma membrane [33, 34], we used PMT-Venus-Rho3^Q74L^ in the assay, which has the first 11 a.a. of Rho3 fused to the N-terminus of Venus-Rho3 [32]. Unfortunately, we can not use full-length Kin2 in BiFC because it displayed autoactivation. The Kin2-C9 (TD2 domain) segment did not autoactivate and displayed an interaction with Rho3^Q74L^ (Fig 7B). The interaction could be detected on the cell cortex. This result suggests that Kin2 and Rho3 may interact *in vivo*. Because Rho3 and Kin2 are both involved in the regulation of exocytosis [6, 7, 31, 35], the interaction between Rho3 and the C-terminal regulatory region of Kin2 raises a possibility that Kin2 could be a potential downstream effector of Rho3 in the regulation of polarized growth *in vivo*. So far, evidence of functional interaction between Kin2 and Rho3 is lacking to support this speculation.

From a two-hybrid screen with full-length Kin2 as bait, we identified N-terminal truncated Bmh1 (region a.a. 5–267) as a protein that interacts with Kin2 (Fig 7C). Bmh1 and its paralog, Bmh2, are two 14-3-3 family proteins. This family of proteins is known to bind phosphorylated proteins and help maintain their structure or localization [36]. Bmh1^5-267^ appears to interact with Kin2-NT, the N-terminal region of Kin2 (Fig 7C, see Kin2-NT and Kin2-NT-KD). This result suggests that Bmh1 may bind Kin2 and regulate Kin2’s activity *in vivo*.

## Discussion

### The localization and targeting mechanism of Kin2

In this study, we show that Kin2 localized to the sites of polarized growth. This localization resembles that of Cdc42, the polarisome, and Sec3 (the spatial landmark for secretion) [28, 37, 38], and fits Kin2’s role in secretion and septin organization. Kin2 is also known to regulate the unfolded protein response in the ER [9]. This finding raises a possibility that Kin2 may localize to the ER. The punctated appearance of GFP-Kin2 on the cell cortex, to some degree, resembles that of the cortical ER network. However, two lines of evidence suggest that Kin2 may not localize to the ER but stays on the plasma membrane. First, the C-terminal segments of Kin2 lacked the punctated appearance and displayed uniform distribution on the cortex (Fig 2B, see Kin2-C8 and Kin2-C9). Second, Kin2’s role in the regulation of ER stress response is solely mediated by the N-terminal kinase domain [9], which we show localized to the bud tip in small-budded cells but not to the entire cell cortex as cortical ER did.

Prior to this study, how Kin2 targets to the membranes is not understood. A previous study suggested that the KA1 domain of Kin2 (here refers to the short Pfam-defined version, 43 a.a. long) plays no role in membrane binding [9]. In this study, we show that Kin2’s localization is mediated by two targeting domains—TD1 and TD2. Both of them could confer localization to the sites of polarized growth but only TD2 could mediate an association with the plasma membrane. The two domains may cooperate for efficient targeting to the sites of polarized growth. The mechanism by which the TD1 domain targets to polarity sites is not known. The TD2 domain (a.a. 882–1147) contains a KA1 domain (a.a. 920–1147), which was delineated based on the sequence alignment with the KA1 domain of mouse MARK3 [19]. The finding that Kin2-TD2 targeted to the plasma membrane is not surprising because KA1 domains in human MARK/PAR-1 kinases such as MARK1 and MARK3 were known to mediate plasma membrane targeting by binding acidic phospholipids [39]. It is likely that Kin2-TD2 may mediate plasma membrane localization by a similar mechanism. In addition to the plasma membrane association, Kin2-TD2 displayed enrichment on the bud cortex. This localization could be due to the asymmetrical distribution of the types of phospholipids to which Kin2-TD2 binds. Alternatively, the interaction of Kin2-TD2 with a yet unidentified protein may contribute to this localization. One example is the *C*. *elegans* PAR-1, which also localizes asymmetrically in the zygote. It was shown that the interaction between the C-terminal region of PAR-1 and NMY-2, the heavy chain of nonmuscle myosin II, is implicated in PAR-1’s asymmetrical localization [40].

Interestingly, sequence alignment of KA1 domains of MARK/PAR-1 kinases from different species showed that the first and last ~40 residues within KA1 domains were highly conserved between animal and fungal orthologs. However, KA1 domains from fungal orthologs often contain additional 10–140 residues between the two conserved regions. For instance, Kin2-KA1 contains 133 additional residues compared to human MARK1-KA1. The extra sequences may play a role in the interaction with other proteins. In the case of Kin2, the additional sequence may contribute to the interactions with Cdc11, Pea2, and Rho3.

### The function and regulation of Kin2

The only two functions of Kin2 known previously are to regulate secretion and the unfolded protein response (UPR) in the ER. In this study, we show that an excess of Kin2 activity impaired septin organization and cell wall structure, suggesting that Kin2 may play extra roles in the regulation of the septin cytoskeleton and the cell wall during bud growth. The fission yeast SpKin1 is known to be implicated in cell wall organization [17]. These findings suggest that fungal orthologs of MARK/PAR-1 may have a general role in the regulation of the cell wall.

**Fig 8:**
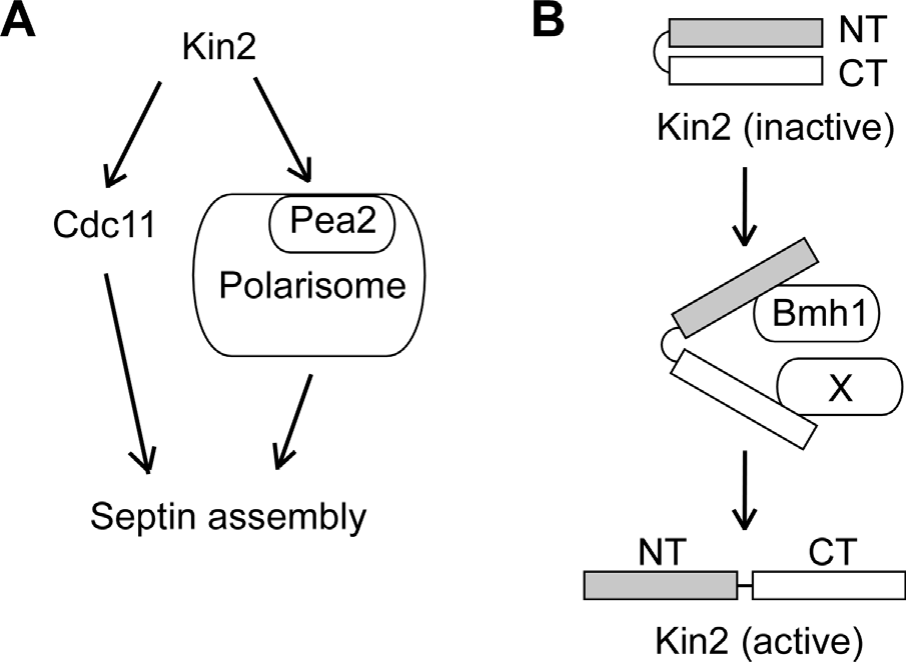
A model for the functional mechanism of Kin2 in septin organization and regulation of Kin2 in the cells. **(A)** Kin2 may play a role in septin organization by interacting with Cdc11 (a septin subunit) and Pea2 (a polarisome component). **(B)** Possible regulation of Kin2. Kin2 is normally in an inactive conformation as the N-terminal region (NT) and C-terminal region (CT) bind each other. The binding of protein X (such as Rho3 or Pea2) to Kin2-CT may help relieve the intramolecular inhibition. The binding of Bmh1 to Kin2-NT may help maintain Kin2 in the active conformation.

The septins are cytoskeletal components. They form an hourglass structure at the mother-bud neck during bud growth, which is critical for the maintenance of a round bud shape [25, 41]. The septin hourglass serves as a diffusion barrier preventing the diffusion of proteins on the daughter side of the plasma membrane into the mother side. It also functions as a scaffold for the anchoring of proteins important for polarized growth and cell cycle progression, such as the chitin synthase III and the protein kinase Hsl1 [41]. The finding that Kin2 is implicated in septin organization is quite unexpected because there are no reports that members in the MARK/PAR-1/Kin1 family regulate septin organization. How could Kin2 regulate septin organization? Our study suggests that both the N-terminal portion and the C-terminal region of Kin2, a.a. 780–1147 (Kin2-C6), are involved in this function. However, the exact mechanisms by which they function in septin organization are not understood at this time. Nonetheless, our observation that the C-terminal region of Kin2 interacted with the septin subunit Cdc11 and the polarisome component Pea2 may provide a clue. The polarisome is a large protein complex that comprises at least Spa2, Pea2, Bud6, and Bni1 [28]. It is known that these components are involved in septin organization during bud emergence as well as during bud growth [29]. We suspect that Kin2-C may regulate septin organization via the interactions with Cdc11 and Pea2 (Fig 8A). The detailed mechanism awaits further investigation.

The kinase activity of Kin2 is thought to be negatively regulated by intramolecular inhibition, in which the C-terminal region of Kin2 binds to the N-terminal region and inhibits its activation [7]. However, it is not understood how the negative regulation is relieved upon Kin2 activation. Particularly, what proteins are responsible for the disruption of this negative regulation. In this study, we identified that Kin2-C interacted with Cdc11, Pea2, and Rho3. This finding raises a possibility that one of these Kin2-C-interacting proteins may be responsible for the activation of Kin2. Because Pea2 and Rho3 share a similar localization with Kin2 [32, 42], we suspect that they may act as the activator for Kin2 by directly binding to the C-terminal region of Kin2 (Fig 8B).

Once the structure of Kin2 is opened up by the binding of an activator to Kin2’s C-terminal region, what molecule may keep the N-terminal region in its activated form? We suspect that the 14-3-3 protein Bmh1 and possibly its paralog, Bmh2 [36], as well, may play such a role. The 14-3-3 family protein PAR-5 is known to function in the same pathway with PAR-1 in the control of anterior-posterior axis in *C*. *elegans* and *Drosophila* [43, 44]. In mammalian epithelial cells, PAR-5 specifically interacts with the phosphorylated form of mammalian PAR-1b/MARK2 [45]. We speculate that the binding of Bmh1 to the N-terminal region of Kin2 may keep Kin2 in its activated conformation and help stabilize the open structure of Kin2 (Fig 8B). Because there is a lack of evidence at this time about the sequential order of the activator and Bmh1 actions, it is possible that Bmh1 may bind to Kin2 before it is activated. Future investigation is needed to address this issue.

As Kin2 orthologs in the human fungal pathogen *Cryptococcus neoformans*, the rice blast fungus *Magnaporthe oryzae*, and the wheat blight fungus *Fusarium graminearum* have been identified as important virulence factors [46, 47], our study will shed light on the investigation of these Kin2 orthologs in pathogenesis.

## Materials and Methods

### Strains, media, and genetic methods

Yeast strains used in this study are listed in Table 1. Standard culture media and genetic techniques were used except where noted. *Escherichia coli* strains DH12S (Life Technologies, Gaithersburg, MD) and DH5α (TaKaRa, Japan) were used as hosts for plasmids manipulation. Yeast cells were routinely grown in SC medium containing 2% glucose (Dex) supplemented with uracil or appropriate amino acids for the maintenance of plasmids. SRG medium that contains 1% raffinose and 2% galactose was used to overexpress genes controlled by galactose-inducible promoters. Oligonucleotide primers for PCR were purchased from Sangon Biotech (Shanghai, China).

**Table 1:** Yeast strains used in this study.

Strain	Genotype	Source
YEF473A	a *his3-*Δ*200 leu2-*Δ*1 lys2-801 trp1-*Δ*63 ura3-52*	[55]
YEF1238	a *his3-*Δ*200 leu2-*Δ*1 lys2-801 trp1-*Δ*63 ura3-52 gin4*Δ::*TRP1*	E. Bi
JGY27B	α *his3-*Δ*200 leu2-*Δ*1 trp1-*Δ*63 ura3-52 sec1-1*	[56]
JGY28B	α *his3-*Δ*200 leu2-*Δ*1 trp1-*Δ*63 ura3-52 sec2-41*	[56]
JGY82A	a *his3-*Δ*200 leu2-*Δ*1 trp1-*Δ*63 ura3-52 sec15-1*	[56]
JGY2030	a *his3-*Δ*200 leu2-*Δ*1 lys2-801 trp1-*Δ*63 ura3-52 swe1*Δ::*HIS3MX*	This study
JGY3084	a *his3-*Δ*200 leu2-*Δ*1 lys2-801 trp1-*Δ*63 ura3-52 kin1*Δ::*KanMX*	This study
JGY3088	a *his3-*Δ*200 leu2-*Δ*1 lys2-801 trp1-*Δ*63 ura3-52 kin1*Δ::*KanMX kin2*Δ::*TRP1*	This study
pJ69-4A	a *his3-*Δ*200 leu2-3*,*112 trp1-901 ura3-52 gal4*Δ *gal80*Δ *LYS2*::*GAL1-HIS3 GAL2-ADE2 met2*::*GAL7-lacZ*	[51]
pJ69-4α	α *his3-*Δ*200 leu2-3*,*112 trp1-901 ura3-52 gal4*Δ *gal80*Δ *LYS2*::*GAL1-HIS3 GAL2-ADE2 met2*::*GAL7-lacZ*	[51]

### Plasmid construction

The pKG21 (2μ *URA3 P*_*KIN2*_-*GFP*-*T*_*CYC1*_) vector was generated for the expression of GFP-tagged Kin2 under the control of *KIN2* promoter. It was constructed by inserting the *Sac*II/*Not*I fragment of *KIN2* promoter (800 bp upstream of the open reading frame) amplified by PCR from genomic DNA, the 728 bp *Not*I-*Bam*HI fragment of *GFP*^S65T^ digested from pKS-GFP, and the 263 bp *Sal*I-*Kpn*I fragment of the *CYC1* transcription terminator from pUG36 [48] into pRS426 (2μ *URA3*). *KIN2* ORF was amplified by PCR from genomic DNA and inserted into *Eco*RI- and *Xho*I-digested pKG21 vector, yielding pKG21-KIN2. For the localization study of Kin2 segments, pUG36 (*CEN URA3 P*_*MET25*_*-yEGFP3-T*_*CYC1*_) was used as the vector. *KIN2* and *KIN2* segments were amplified by PCR from genomic DNA and inserted into the *Eco*RI- and *Xho*I-digested pUG36, yielding pUG36-KIN2 and pUG36-KIN2 segments, respectively. pRS315-GFP-RAS2 that expresses GFP-Ras2 was described previously [49].

For the multicopy suppression of temperature-sensitive *sec* mutants, we first generated pRS426-*T*_*CYC1*_ (2μ *URA3 T*_*CYC1*_) vector by inserting the *Sma*I-*Kpn*I fragment of the *CYC1* transcription terminator from pUG36 into pRS426 (2μ *URA3*). Then, *KIN2* and *KIN2* N-terminal segments with 800 bp *KIN2* promoter were amplified by PCR from genomic DNA and inserted into *Eco*RI- and *Xho*I-digested pRS426-*T*_*CYC1*_, yielding pRS426-KIN2 and pRS426-KIN2 segments.

For *GAL*-driven overexpression of *KIN2* and *KIN2* segments, *Eco*RI-*Xho*I fragments of *KIN2* and *KIN2* segments were digested from pUG36-KIN2 and pUG36-KIN2 segments and inserted into *Eco*RI- and *Sal*I-digested pEGKT316 (*CEN URA3 UAS*_*GAL1*_-*P*_*CYC1*_-*GST*-*T*_*CYC1*_) [50].

For yeast two-hybrid assay, *Eco*RI-*Xho*I fragments of *KIN2* segments were digested from pUG36-KIN2 segments and inserted into *Eco*RI- and *Sal*I-digested pGBDU-C1 (2μ *URA3 GAL4-DBD*) [51], yielding pGBDU-KIN2 segments. pGAD-PEA2 was generated by inserting the *Sma*I-*Sal*I fragment of *PEA2* ORF into pGAD-C1 (2μ *LEU2 GAL4-AD*) [51]. pGBDU-RHO3^Q74L, ΔC^ and pGBDU- RHO3^T30N, ΔC^ were described previously [32].

For bimolecular fluorescence complementation (BiFC) assay, plasmid vectors pVN1 (*CEN URA3 P*_*MET25*_-*Venus*-*N*-*T*_*CYC1*_) and pVC1 (*CEN HIS3 P*_*MET25*_-*Venus*-*C*-*T*_*CYC1*_) were used [52]. pVC1-KIN2-C6 and pVN1-KIN2-C9 were generated by inserting the *EcoR*I-*Xho*I fragments of *KIN2-C6* (encoding a.a. 780–1147) and *KIN2-C9* (encoding a.a. 882–1147) into pVC1 and pVN1, respectively. pVN1-PEA2 was generated by inserting the *Sma*I-*Sal*I fragment of *PEA2* ORF into pVN1. An *Eco*RI-*Sal*I fragment of *RHO3*^*Q74L*^ was inserted into pVC1, yielding pVC1-RHO3^Q74L^. The *P*_*MET25*_ promoter in pVC1-RHO3^Q74L^ was replaced with *P*_*MET25*_*-PMT* (*PMT* encodes the first 11 a.a. of Rho3) [32], yielding pVC1-PMT-RHO3^Q74L^.

### Yeast strain construction

*KIN1* was deleted in YEF473A by a PCR-based method [53], yielding strain JGY3084 (a *kin1*Δ::*KanMX*). *KIN2* was deleted in JGY3084 by the same method, yielding strain JGY3088 (a *kin1*Δ::*KanMX kin2*Δ::*TRP1*). Likewise, *SWE1* was deleted in YEF473A, yielding JGY2030 (a *swe1*Δ::*HIS3MX*). YIp128-CDC3-GFP [54] was linearized by *Bgl*II for integration at the *CDC3* locus in yeast, yielding the integrated *CDC3-GFP*:*LEU2* allele.

### Yeast two-hybrid screen

The three screens were performed in the yeast strain pJ69-4A or pJ69-4α using a pOAD-cDNA prey library. In the first screen, pGBDU-KIN2 was used as the bait. Transformants were grown on SC-Leu-Ura-His plates containing 3 mM 3-AT at 30°C. The plates were then replica plated onto SC-Leu-Ura-Ade plates to allow the identification of candidate clones. The pOAD-prey library plasmids were retrieved and the cDNA inserts were sequenced. We screened 340,000 transformants and isolated two positive clones. They encode N-terminally truncated Bmh1 (a.a. 5–267) and Irc8 (a.a. 377–822). In the second screen, pGBDU-KIN2-Δ42 was used as the bait. 423,600 transformants were screened and 44 positive clones were isolated. One clone encodes Cdc11 (a.a. 72–415). Two clones encode Tos1 (a.a. 192–455) and Tos1 (a.a. 201–455). The third screen used pGBDU-RHO3^Q74L,ΔC^ as the bait. cDNA clones isolated from this screen that encode N-terminally truncated Rga1, Myo2, and Exo70 were described previously [32]. One cDNA clone encodes Kin2 (a.a. 1011–1147).

### Yeast two-hybrid assay

Cells of strain pJ69-4α carrying the pGBDU-C1-based (2μ *URA3 GAL4-DBD*) bait plasmids were mated with cells of strain pJ69-4A carrying the pOAD-based (*CEN LEU2 GAL4-AD*) or pGAD-based (2μ *LEU2 GAL4-AD*) prey plasmids on YPD plates and then replica plated onto SC-Ura-Leu plates to select for diploid cells that harbor both bait and prey plasmids. Diploid cells were replica plated onto SC-Leu-Ura-His or SC-Leu-Ura-Ade to check for growth. Growth indicates interaction between the DBD and AD fusion proteins.

### Microscopy

An Olympus BX51 microscope (Tokyo, Japan) and a Retiga 2000R CCD camera (QImaging Corporation, Canada) were used in the visualization of cell morphology and GFP-tagged proteins by differential interference contrast (DIC) and fluorescence microscopy. The images were obtained using QCapture Suite (QImaging Corporation, Canada). ImagePro Plus (Glen Mills, PA) was used for image processing. To visualize the distribution of chitin in the cell wall, yeast cells were stained with 0.01% calcofluor white (Sigma-Aldrich, USA) for 5 min.

## Supporting Information

S1 Fig Expression of GFP-Kin2 segments in yeast cells. Cells of strain YEF473A carrying pUG36-KIN2 segments were grown in SC-Ura medium. Cell lysates were prepared and the proteins were separated by 7.5% SDS-PAGE and immunoblotted with anti-GFP antibody. Molecular weight of GFP-fusion proteins: GFP-Kin2 (FL, 154 kDa), GFP-Kin2-Δ42 (149 kDa), GFP-Kin2-NT (85 kDa), GFP-Kin2-N7 (84 kDa), GFP-Kin2-N5 (83 kDa), GFP-Kin2-N7ΔN (73 kDa), GFP-Kin2-C6 (68 kDa), GFP-Kin2-C8 (61 kDa), GFP-Kin2-C9 (57 kDa), and GFP-Kin2-C10 (53 kDa). GFP (238 a.a. plus linker 12 a.a., 27.5 kDa). Note: Kin2-C6 and Kin2-C8 segments migrated slower than predicted.
(TIF)
