# Supplementary figures and images for: Kin2, the Budding Yeast Ortholog of Animal MARK/PAR-1 Kinases, Localizes to the Sites of Polarized Growth and May Regulate Septin Organization and the Cell Wall
Source: PLoS One. 2016 Apr 20;11(4):e0153992. doi: 10.1371/journal.pone.0153992 (PMC4838231; doi:10.1371/journal.pone.0153992)

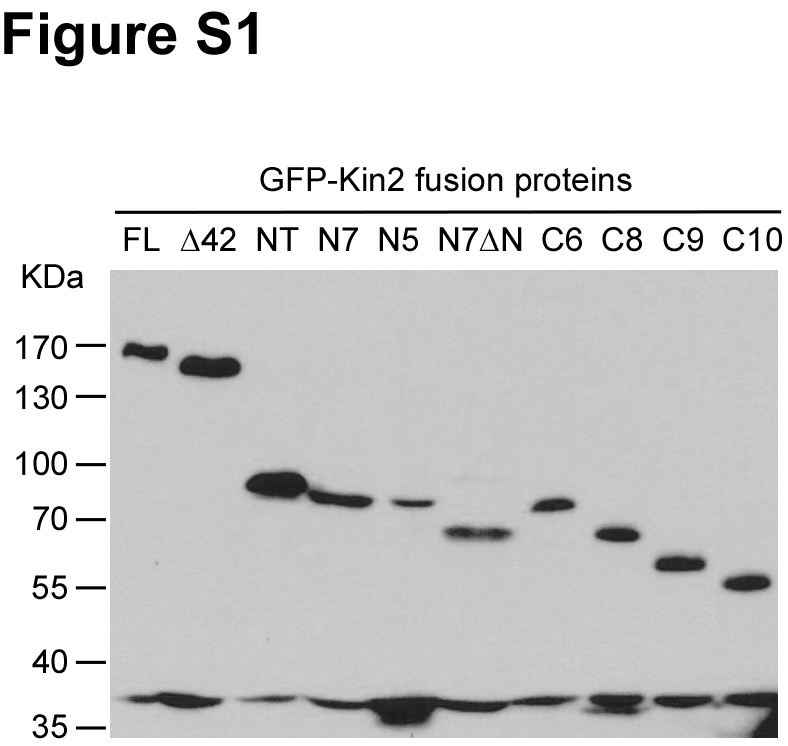

Supplement: S1 Fig — Cells of strain YEF473A carrying pUG36-KIN2 segments were grown in SC-Ura medium. Cell lysates were prepared and the proteins were separated by 7.5% SDS-PAGE and immunoblotted with anti-GFP antibody. Molecular weight of GFP-fusion proteins: GFP-Kin2 (FL, 154 kDa), GFP-Kin2-Δ42 (149 kDa), GFP-Kin2-NT (85 kDa), GFP-Kin2-N7 (84 kDa), GFP-Kin2-N5 (83 kDa), GFP-Kin2-N7ΔN (73 kDa), GFP-Kin2-C6 (68 kDa), GFP-Kin2-C8 (61 kDa), GFP-Kin2-C9 (57 kDa), and GFP-Kin2-C10 (53 kDa). GFP (238 a.a. plus linker 12 a.a., 27.5 kDa). Note: Kin2-C6 and Kin2-C8 segments migrated slower than predicted. (TIF) [file pone.0153992.s001.tif]
